# Effects of Oral Antihistamines on Tear Volume, Tear Stability, and Intraocular Pressure

**DOI:** 10.3390/vision4020032

**Published:** 2020-06-20

**Authors:** Brian K. Foutch, Kyle A. Sandberg, Edward S. Bennett, Leonard L. Naeger

**Affiliations:** 1 Rosenberg School of Optometry, University of the Incarnate Word, San Antonio, TX 78229, USA; KSandber@uiwtx.edu; 2 College of Optometry, University of Missouri-St Louis, St. Louis, MO 63121, USA; EBennett@umsl.edu (E.S.B.); bbfoutch@gmail.com (L.L.N.); 3 St. Louis College of Pharmacy, St. Louis, MO 63110, USA

**Keywords:** tear break-up time, phenol red thread test, intraocular pressure, antihistamine, diphenhydramine, loratadine

## Abstract

The goal of this study was to investigate the effects of two commonly used oral antihistamines—diphenhydramine and loratadine—on tear volume, tear breakup time, and intraocular pressure. Placebo, diphenhydramine, and loratadine were administered for one week to 33 subjects experimentally blind to the treatment given. All the subjects received all three treatments over a period of six weeks. The outcome measures were the change in phenol red thread test (PRT), the tear breakup time (TBUT), and the intraocular pressure (IOP) of both eyes evaluated by experimentally masked observers. Neither of the mean changes in TBUT or IOP depended on the treatment given, but there was a significant monocular decrease in tear volume from diphenhydramine use. While we used an adequate treatment washout period of seven days, our investigation was limited by the short treatment times and inclusion of only young healthy patients. Overall, however, these results suggest that systemic diphenhydramine use should be limited to increase the effectiveness of conventional therapies. Clinicians should have fewer reservations about recommending the use of loratadine concurrent with dry eye treatments.

## 1. Introduction

Many systemic medications can alter tear production and composition by antagonizing muscarinic (or M-) receptors. M-receptors, particularly of the M_3_ subtype, are cholinergic receptors [1] that help to regulate fluid secretion in the eye by acting on the lacrimal gland and the ciliary body. The activation of M-receptors also stimulates conjunctival goblet cells to produce mucin [1]. Blocking these receptors can then cause abnormal aqueous or mucin layers and can lead to tear film instability, often resulting in ocular discomfort. Antihistamines have such an anti-cholinergic activity, and patients do often complain of their drying effect on the eyes.

Two commonly used oral antihistamines (OAs) for allergic rhinitis or conjunctivitis are diphenhydramine and loratadine. Diphenhydramine HCl was first approved by the Federal Drug Administration (FDA) in 1946 under the brand name Benadryl [2]. Benadryl is still marketed as an over-the-counter allergy and hay fever medication, but generic diphenhydramine is also sold under other trade names and is commonly used as an ingredient in many cold and allergy remedies. Due to its non-polar nature, diphenhydramine can have significant central nervous system (CNS) side-effects [3]. These include dystonia [4,5,6], dyskinesias [7,8], dry mouth or eyes [9,10], and mild sedation/drowsiness [3,11,12]. It has also been long-established that the CNS effects of diphenhydramine are related to dosage [13], and the recommended dosages of 25–50 mg given every 4 to 6 h vary in their toxicity [2].

Loratadine, an orally administered polar antihistamine with far fewer CNS effects than diphenhydramine, has been available for over-the-counter use since 2002 [2]. Consequently, there have been many investigations implicating the drying effects of loratadine [14,15,16,17], but we are aware of no randomized controlled clinical trials directly comparing the drying effects of diphenhydramine and loratadine in normal subjects. 

Intraocular pressure (IOP) depends on the steady state of production and drainage of the aqueous humour in the anterior chamber of the eye. Many oral antihistamines have been implicated in acute angle closure attacks in patients with narrowed aqueous circulation (e.g., narrow-angle glaucoma) [18,19]. There is a paucity of information on their effects on IOP in humans, but a few animal studies have associated antihistamines with reduced IOP [20,21,22].

The objective of this study was to investigate the effects of these two commonly used oral antihistamines at normal doses—diphenhydramine (25 mg, three times per day) and loratadine (10 mg, once per day)—on tear volume, tear breakup time, and intraocular pressure.

## 2. Materials and Methods

### 2.1. Subjects

Thirty-three subjects participated, and the ages ranged from 22–28 years. The exclusion criteria included pregnant women (or those planning pregnancy); any use of prescribed or over-the-counter (OTC) antihistamine, anticholinergic, or cold remedies; any current use of any topical eye medications; any cognitive impairment; and any systemic condition or ill health. We limited our subject pool to healthy participants to avoid the potential confounding or interaction of any anterior segment ocular disease with the antihistamine treatment. As our subjects were recruited by convenience at a health profession training program, our volunteers tended to be younger than if we had performed this investigation in a different setting. The protocol was approved by the Institutional Review Board at the University of Missouri-St Louis (#030213C; 28 Feb 2003), and informed consent was obtained from each subject.

### 2.2. Treatment Preparation

All the treatments were prepared in #1 capsules (Qualicaps^®^, Inc., Whitsett, NC, USA). Placebo capsules were packed with lactose in the typical manner. For diphenhydramine, 25 mg capsules (Qualitest [Endo] Pharmaceuticals, Newark, DE, USA) were opened and the contents placed into #1 capsules. For loratadine, 10 mg tablets (Geneva Pharmaceuticals, Inc., Broomfield, CO, USA) were placed in #1 capsules partially filled with lactose (NF hydrous; Professional Compounding Centers of America, Inc., South Jordan, UT, USA). For diphenhydramine and loratadine, lactose was used to fill the remainder of the capsules to disguise the presence of the drug present.

Cold seal dosage cards (Drug Package, Inc., O’Fallon, MO, USA)—each representing a seven-day supply with AM, noon, and 6 PM dose slots—were filled with placebo (three capsules/day), diphenhydramine (three capsules/day), or loratadine (loratadine in the AM slot and placebo in the remaining two slots). The sequence in which the cards were taken was varied, but each participant received one card for each of the three dosage regimens.

### 2.3. Experimental Design

After the subjects were determined eligible, they each randomly selected an identifying number from a bag. Each subject also had one of six protocol identifiers (from 1 through 6) assigned to them at random. The protocol numbers were not equally distributed among the 33 subjects and corresponded to the following order of the treatments (where A = loratadine, B = diphenhydramine, and C = placebo; [ ] = # of subjects): 1 = ABC [8], 2 = ACB [3], 3 = BAC [4], 4 = BCA [6], 5 = CAB [5], and 6 = CBA [7]. For example, a subject assigned Protocol 1 (i.e., ABC) underwent the following schedule:
Day 0Subjective (eligibility) and objective evaluation.Day 1–7Loratadine 10 mg upon waking in AM, placebo at noon, placebo at 6 PM.Day 8Objective evaluation.Day 8–14Washout period.Day 14Objective evaluation.Day 15–21Diphenhydramine 25 mg upon waking in AM, at noon, and at 6 PM.Day 22Objective evaluation.Day 22–28Washout period.Day 28Objective evaluation.Day 29–35Placebo upon waking in AM, noon, and at 6 PM.Day 36Objective evaluation.

### 2.4. Objective (Clinical) Measures

Phenol red thread (PRT) testing, tear break-up time (TBUT) using fluorescein dye, and intraocular pressure (IOP) evaluation were then performed in that order on each eye of each subject six times (one week apart) by qualified investigators masked to the current treatment. 

#### 2.4.1. Phenol Red Thread Test

While the phenol red thread tear test (ZONE-QUICK, Showa Yakuhin Kako Co., Ltd., Chuo-Ku, Tokyo 104, Japan) has been shown to have low diagnostic value by itself for dry eye disease [23], it takes little time to administer, causes minimal discomfort, and has been shown to be an adequate measure of residual tear volume in the inferior conjunctiva [24]. The thread is acidic and yellow in color until it contacts tears, which cause a color change to light red. Forceps were used to insert the thread’s 3 mm folded portion into the lower palpebral conjunctiva of each eye approximately 1/3rd of the distance from the lateral canthus. After 15 s, the thread was removed, and the red portion was measured.

#### 2.4.2. Tear Break-Up Time

The assessment of tear break up time (TBUT) with fluorescein is a benchmark clinical measure of tear stability (reviewed by McMonnies [25]). It is effectively the rate of tear loss by evaporation and captures the additive roles of aqueous, mucin, and lipid deficiencies in tear instability [26,27,28]. After fluorescein was administered to each eye, the investigator viewed the tear film and instructed the subject to blink a few times and then stare straight ahead. The time (in seconds) from the last blink until the tear film integrity is visibly disrupted was recorded as the TBUT.

#### 2.4.3. Intraocular Pressure

We measured the intraocular pressure in each eye using Goldmann applanation [29]. This technique required the use of a topical anesthetic and fluorescein but is minimally invasive, takes little time to administer, and is considered the gold standard clinical measure of IOP [30].

### 2.5. Data Analysis

We first performed a descriptive analysis of the raw PRT, TBUT, and IOP values. The dependent variables in all our primary analyses were the change in PRT, TBUT and IOP measures for the right and left eyes. We performed a repeated-measures analysis of variance (ANOVA) across all six dependent variables, with the protocol sequence as a between-subjects factor (with Tukey honest significant difference (HSD) applied for multiple comparisons by the protocol sequence). We then performed post-hoc *t*-tests on the change variables, comparing the left and right eyes. Lastly, we compared the changes in PRT, TBUT, and IOP for the right and left eyes separately. *p*-values < 0.05 were considered statistically significant. Excel (Microsoft Corp., Redmond, WA, USA) and SPSS (IBM, Chicago, IL, USA) were used for all the statistical analyses.

## 3. Results

### 3.1. Descriptive Results

The PRT values across all the conditions and averaged for both eyes ranged from 7 to 35 mm, with a mean (±SD) of 21.42 (±5.90). The median PRT value (averaged for both eyes) was 22.0 mm. The TBUT values averaged for both eyes ranged from 4 to 22 s, with a mean (±SD) of 8.68 (±2.88). The median TBUT value (averaged for both eyes) was 9.0 mm. The IOP values averaged for both eyes ranged from 9 to 21 mmHg, with a mean (±SD) of 14.89 (±3.81). The median IOP value (averaged for both eyes) was 15.0 mmHg. The mean values were equivalent between the right and left eyes for PRT (*t*[65] = 0.562, *p* = 0.576), TBUT (*t*[65] = −0.050, *p* = 0.944), and IOP (*t*[66] = 0.259, *p* = 0.796). The descriptive PRT, TBUT, and IOP results are shown for the right and left eyes separately in Table 1.

### 3.2. Changes in Tear Volume

**Figure 1:**
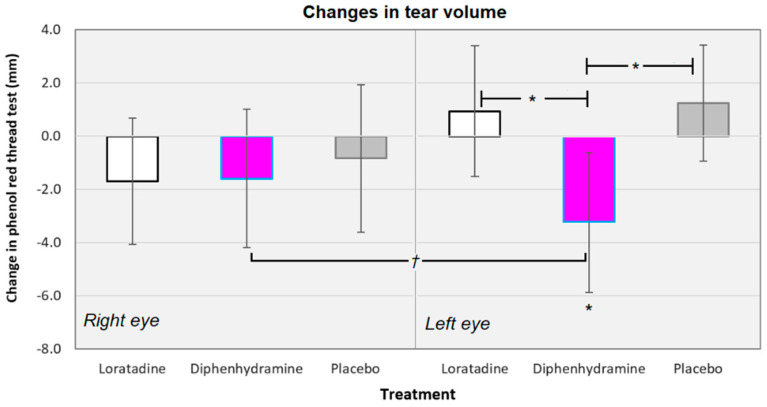
Changes in the tear volume averaged across all the subjects for the right and left eyes, separately. There were no significant differences between treatments for the right eye, but diphenhydramine significantly reduced the tear volume in the left eye. There was a trend for diphenhydramine use to reduce the tear volume when averaged for the right and left eyes (*p* = 0.072). Diphenhydramine also reduced the tear volume in the left eye more than the placebo and loratadine. * *p* < 0.05, ^†^ *p* < 0.10; Error bars represent ±95% confidence intervals.

Across all conditions, there was a −0.88 mm mean decrease in PRT values (−1.42 mm when not considering the placebo conditions). The mean changes in PRT values ranged from −6.80 mm (for loratadine use, left eye, subjects were assigned to CAB protocol) to +4.67 mm (for loratadine use, right eye, subjects assigned to BAC protocol). The changes in tear volume were equivalent across all the six treatments (*F*[5130] = 1.796, *p* = 0.179; see Figure 1). The changes in PRT values were equivalent between the right and left eyes for the loratadine (*t*[65] = 1.545, *p* = 0.127), diphenhydramine (*t*[65] = 0.939, *p* = 0.351), and placebo (*t*[65] = 1.193, *p* = 0.237) conditions.

Diphenhydramine did reduce the tear volume in the left eye by an average of −3.33 mm, a statistically significant reduction (*t*[31] = −2.425, *p* = 0.021). There was a trend for diphenhydramine use to reduce PRT values when averaged for right and left eyes together (*t*[31] = 1.864, *p* = 0.072). In addition, diphenhydramine reduced the tear volume more than the placebo (mean difference = −4.58 mm; *t*[62] = −2.652, *p* = 0.010) and loratadine (mean difference = 4.27 mm; *t*[62] = −2.345, *p* = 0.022; see Figure 1).

**Figure 2:**
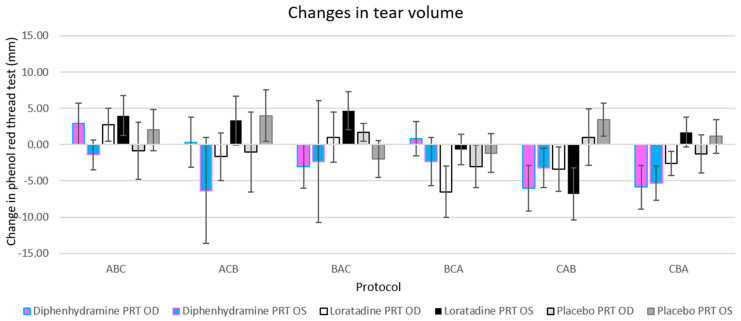
Changes in tear volume were equivalent when compared by protocol sequence. The only treatment that reduced the tear volume for all protocols was diphenhydramine in the left eye (light blue bars), but a post-hoc analysis (via Tukey honest significant difference (HSD)) showed no significant changes for multiple (six) treatments within each protocol. Error bars represent ±1 standard error of the mean (SEM).

The changes in tear volume were equivalent across treatments when compared by protocol sequence (*F*[5,26] = 1.614, *p* = 0.192; see Figure 2). In addition, there were no differences in tear volume changes between protocol sequences within each treatment when controlled for multiple comparisons by Tukey honest significant difference (HSD). The only consistent finding across protocols was the reduction in tear volume from diphenhydramine in the left eye, but no significant treatment/protocol combination resulted in a significant reduction or decrease in tear volume (with Tukey HSD correction applied; see Figure 2).

### 3.3. Changes in Tear Stability

**Figure 3:**
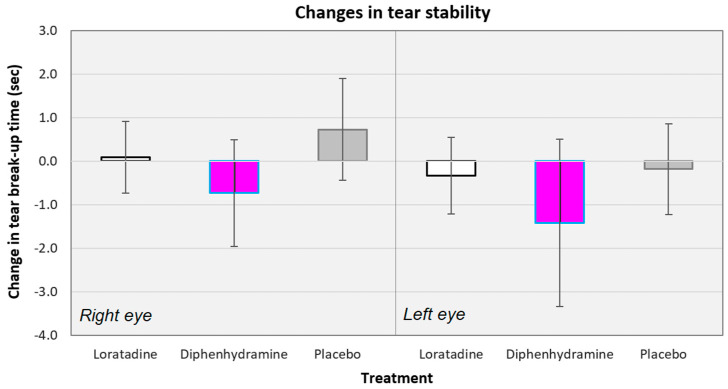
Changes in tear break-up times averaged across all subjects for the right and left eyes, separately. There were no significant differences between the treatments for either eye. Error bars represent ±95% confidence intervals.

Across all conditions, there was a −0.31 s mean decrease in the TBUT values (−0.60 s when not considering the placebo conditions). The mean changes in TBUT values ranged from −4.67 s, (for diphenhydramine use, left eye, subjects assigned to BAC protocol) to +4.67 s (for loratadine use, left eye, subjects assigned to ACB protocol). The changes in tear stability were equivalent across all the treatments (*F*[5135] = 0.849, *p* = 0.517; see Figure 3). Diphenhydramine did reduce the mean tear break-up times in the right (−0.73 s; *t*[32] = −1.164, *p* = 0.253) and left (−1.42 s; *t*[32] = −1.451, *p* = 0.157) eyes, but neither reduction was significant. The changes in TBUT values were equivalent between the right and left eyes for the loratadine (*t*[66] = 0.693, *p* = 0.491), diphenhydramine (*t*[66] = 0.602, *p* = 0.549), and placebo (*t*[66] = 1.550, *p* = 0.252) conditions.

The changes in tear break-up time were also equivalent across the treatments when compared by protocol sequence (*F*[5,27] = 0.763, *p* = 0.584; changes in TBUT across protocol sequences not shown). There were also no differences in tear stability changes between the protocol sequences within each treatment when controlled for multiple comparisons by Tukey HSD. There were no consistent increases or decreases in tear stability for any treatment across protocols.

### 3.4. Changes in Intraocular Pressure

**Figure 4:**
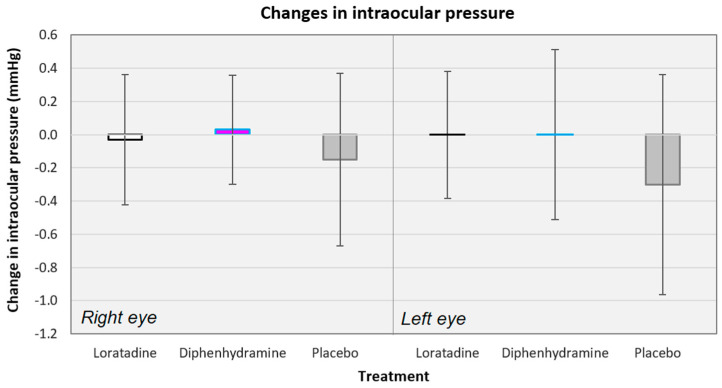
Changes in the intraocular pressure averaged across all subjects for the right and left eyes, separately. There were no significant differences between treatments for either eye. Error bars represent ±95% confidence intervals.

Across all conditions, there was only a −0.08 mmHg mean decrease in the IOP values (0.00 mmHg when not considering the placebo conditions). The mean changes in IOP ranged from −1.29 mmHg (for diphenhydramine use, left eye, subjects assigned to CBA protocol) to +1.00 mmHg (for diphenhydramine use, left eye, subjects assigned to ACB protocol). The placebo conditions showed the largest decrease in IOP when averaged across protocols (see Figure 4). The changes in IOP were equivalent across all the treatments (*F*[5135] = 0.556, *p* = 0.734) and across the treatments when compared by protocol sequence (*F*[5,26] = 0.625, *p* = 0.682; changes in IOP across protocol sequences not shown). There were also no differences in the IOP changes between protocol sequences within each treatment nor consistent increases or decreases in IOP for any treatment across protocols. Lastly, the mean changes in IOP were equivalent between the right and left eyes for the loratadine (*t*[65] = 0.110, *p* = 0.913), diphenhydramine (*t*[65] = 0.098, *p* = 0.922), and placebo (*t*[65] = 0.356, *p* = 0.723) conditions.

## 4. Discussion

It is well established that there is a symptomatic and objective link between oral antihistamines (OAs) and ocular dryness [15,16,17,22]. The goal of this study was to link clinical signs with two commonly used OAs, diphenhydramine and loratadine. Furthermore, IOP monitoring was performed in our subjects, as previous evidence has supported some potential effects of these drugs on aqueous production as well as their potential to induce angle closure attacks through their anticholinergic activity [18,19]. Conversely, animal models have demonstrated no effect on IOP or even IOP reduction in response to antihistamine administration [20,21,22]. The monitoring of IOP in our subjects was therefore a natural extension of this study’s objectives.

While the primary goal of this study was to evaluate the changes in tear volume, tear stability, and intraocular pressure, we did verify that the mean values in our subjects for PRT (21.42 ± 5.90 mm) and IOP (14.89 ± 3.81 mmHg) fell within previously reported population norms [23,24,27,28,30]. Our mean TBUT values (8.68 ± 2.88 s) may seem surprising, as TBUT values of less than 10 s are considered by many to be diagnostic of lipid layer dysfunction [27]. However, there are many studies that find average TBUT values of as low as 5–9 s in healthy eyes [25].

A key takeaway in our study was that we found no significant change from baseline in tear volume, tear stability, or IOP in any of the groups when using either the placebo or loratadine. The treatment order made no significant difference to these findings. The lack of impact of loratadine on the tears of our normal subjects represents a finding that contradicts much, but not all, of the literature. The second key finding was that diphenhydramine showed a significant decrease in tear volume in the left eye of our subjects when compared to the placebo (mean difference = −4.58 mm; *t*[62] = −2.652, *p* = 0.010) and loratadine (mean difference = 4.27 mm; *t*[62] = −2.345, *p* = 0.022). There was also a marginally significant decrease in the mean tear volume averaged for both eyes with diphenhydramine (*t*[31] = 1.864, *p* = 0.072). These findings are consistent with a classic case of filamentary keratitis in a patient with severe keratoconjunctivitis sicca treated with artificial tears, nighttime lubrication, bacitracin ointment, and a separate antibiotic-steroid combination and severe allergic rhinitis treated with several systemic medications including high doses (as much as 300 mg/day) of diphenhydramine [9]. All the systemic antihistamines were discontinued, and the patient recovered normal tear functioning with hourly artificial tear use. However, the filaments returned after the patient started self-medicating with relatively high doses (up to 200 mg/day) of diphenhydramine. Our monocular findings were curious, and a potential explanation is offered in the discussion on limitations below. Diphenhydramine did not significantly affect the tear stability (TBUT) nor IOP in any treatment group.

While many studies have shown that dry eye findings are more common in patients using OAs, the evidence is equivocal. Moss and colleagues examined the risk factors for the prevalence of dry eye in the Beaver Dam Eye Study cohort (*n* = 3722) and found that oral antihistamines were not a causative factor for dry eyes [31]. However, these results were based solely on dry eye symptom reporting and did not rely on any clinical measurements. Further, Moss and colleagues published before loratadine was available over the counter, which occurred in November 2002 [2,32]. In our study, the addition of loratadine to the protocol as a dependent variable offers a relevant update to this information. Loratadine has been heavily marketed under the trade name Claritin and recently ranks 2nd in terms of sales of oral antihistamines in the US [33,34]. While there have been other OTC antihistamine products added to the market since the time our data were collected, loratadine sold in formulations both in branded forms such as Claritin and in-house brands continues to be an extremely popular choice among the general public for treatment of allergy. As a class of medications, a recent Nielsen study showed that as antihistamines have become largely available without a prescription, more patients are turning to over-the-counter remedies [35]. This same study showed that although health care providers wrote 31% fewer allergy prescriptions over a six-year period, nearly half of all patients stated that a health care provider recommendation was an influence on their over-the-counter medication choice. This point highlights the important role eye care professionals play in patient medication selection and offers an area for potential modification of clinical practice.

Additional support for our findings that loratadine has no effect on tear volume comes from Kellerman, who examined a group of 222 patients already enrolled in a dry eye clinical trial [14]. The study found that patients that entered the trial on oral antihistamines (including loratadine) did not show any difference in corneal staining, Schirmer scores, or TBUT from non-antihistamine users at baseline; however, there was greater conjunctival staining observed in the antihistamine group. This data may impact clinical practice, as described below.

### 4.1. Strengths

The primary strength of our study was the robust design. The study group assignments were randomized, and the data collection was double masked and placebo controlled. The clinical testing was straightforward and repeatable, leaving little room for misinterpretation. By using a highly consistent drug dosing and delivery method throughout the study period, we ensured that the participants would not be biased by their oral medication protocol. The length of the washout period between treatments also contributes to the validity of the findings. With a minimum seven-day washout period, it is reasonable to assume that both diphenhydramine and loratadine would be fully eliminated from the systemic circulation of healthy subjects, considering their respective half-lives of 2.4–9.3 h and 3–20 h [36,37]. This aspect essentially removes the possibility of the cross-therapy contamination of the results.

### 4.2. Limitations

An obvious limitation of our study is the small number of subjects (*n* = 33). We were asking a lot of our subjects in terms of participation time, but we were also forced to consider our time and resources and fixed our subject pool to 30–36 participants in lieu of a formal a priori power analysis. Using a conservative medium effect size of d = 0.40, we would have required 100–200 subjects for a power (1-β) of 0.80 via *t*-tests with two-tailed comparisons. That number of subjects would have been impractical. A post hoc power analysis did not yield significantly different results. For our one significant positive finding of reduced tear volume in the left eye with diphenhydramine use, the effect size (Cohen’s d) was 0.445, or medium. The effect size for our marginally significant finding of reduced tear volume (both eyes averaged) with diphenhydramine use was d = 0.333, also a medium effect. Therefore, we found a medium effect size but acknowledge that these comparisons were under-powered and possibly limit inferences from our study.

In addition, our protocol did not establish that tear volume assessments would be randomized, which represents a limitation in our study design. This perhaps explains the unexpected finding in our trial that only the left eye of our patients taking diphenhydramine showed any significant change in tear volume vs. placebo and loratadine. The postulate is that perhaps the examiners routinely started with the left eye when testing tear volume. While it is conventional in eye care to test the right eye first, factors such as examiner handedness and the room layout and its influence on access to materials or subject preference may have caused examiners to deviate from this convention. If the left eye was routinely tested first, it may account for some bilateral reflex tearing, as PRT has been shown to cause some degree of reflex tearing [38]. This could account for the higher tear volume measured in the right eye. Unfortunately, the eye that was tested first was not recorded in our data collection, allowing only speculation when attempting to explain the difference in the right and left eye findings.

Perhaps another test of tear volume that does not produce nor measure reflex tears (e.g., Schirmer’s Type 2) would have been a more appropriate measure. However, the phenol red thread (PRT) test was developed to overcome the variability and poor repeatability of Schirmer’s testing. In addition, the test time of only 15 s in comparison to 5 min per eye needed for Schirmer testing made PRT more appropriate in our investigation, where we already asked for a lot of participants’ time. Thus, while it has also been argued that PRT really measures differences in absorption by the thread and not the residual volume, [23] we felt it was most appropriate for our purposes.

Another limitation was the lack of comparison of a healthy cohort with one that is experiencing ocular or systemic allergy. These patients are the most likely to be routine antihistamine users, and their inclusion in a future trial could help to unlock some of the mechanisms behind ocular dryness as it relates to the oral administration of these drugs.

Lastly, while we are convinced that our washout periods were appropriate for sufficient drug clearance between treatments, our design does not at all address the potential consequences of long-term or chronic usage of over-the-counter antihistamines. This could explain some of our negative findings. Future designs could do so through longer-term (i.e., 6–9 month) cross-over designs, where groups are exposed to 2–3-month treatments.

### 4.3. Clinical Extensions

With as many as 60 million people suffering from allergic rhinitis in the US alone [39], the average eye care provider is likely to see patients on OA therapy. The lack of a correlation between OAs and tear stability and tear volume in most of our test scenarios is encouraging evidence in the treatment of systemic and ocular allergy. This suggests that the eye care practitioner should feel more confident in recommending these treatment options to patients without the fear of inducing unwanted drying effects. This is especially true when recommending loratadine. It may be reasonable to infer that other drugs that work with a similar mechanism of action to this second-generation antihistamine would have similar results, although this represents an area that warrants further investigation. Direct comparison between other commonly used second-generation antihistamines such as levocetirizine, cetirizine, and fexofenadine may result in evidence that one drug outperforms the others in terms of unwanted ocular side effects.

In the treatment of tear film irregularities in atopic patients, it is important to consider that the disease itself (allergic conjunctivitis) may be the primary cause of dryness due to its recognized impact on ocular lipid composition [40]. This postulate would suggest that previous investigations into the pathophysiology of OA-induced dryness are flawed. Our data helps to support this supposition and further confirmation of this model may suggest that second-generation antihistamine medications can be safely continued while the ocular surface disease is addressed with conventional treatments. However, further research into this relationship is needed before clinicians routinely recommend oral antihistamine use in the setting of significant dry eye.

Our experiment did show a significant decrease in tear volume in patients treated with diphenhydramine, suggesting that the discontinuation or avoidance of this medication might be indicated in the setting of dry eye. With a wide variety of effective choices available and considering the known CNS side effects of diphenhydramine, the clinician should consider counseling patients on alternative systemic allergy therapies. It should further be recognized that diphenhydramine is a common ingredient in sleep aids such as Tylenol PM, Advil PM, and Unisom. The amount of diphenhydramine in these medications is as much as double the recommended single dosage for routine allergy treatment (25 mg/dose vs. 50 mg/dose) [41,42] and may represent an even greater risk for the development of ocular symptoms. These facts highlight the importance of routine and thorough questioning of patients on their over-the-counter medication usage.

A final clinical take-home is that we did not demonstrate a change from the baseline IOP in any of our groups, regardless of the order in which the interventions were applied. This should give the clinician confidence that IOP will not be affected in normal patients when recommending any type of OA. We did not study this relationship in patients with glaucoma, however, and we cannot comment on the effects of OAs in this population.

## 5. Conclusions

The link between oral medication usage and ocular signs and symptoms is well recognized across a myriad of drug classes. Oral antihistamines represent one of the most commonly used categories of drugs in the US, with 75 percent of allergy sufferers treating their symptoms with over-the-counter remedies [41]. Our data showed that there was a significant decrease in tear volume in the left eye and a marginal decrease when averaged for both eyes among our subjects taking diphenhydramine. This effect was not observed in any other treatment group. This decrease was not present when we measured TBUT in any treatment group, including diphenhydramine. Finally, there was no significant change in IOP from baseline in any treatment group. When treating patients for ocular dryness, these results suggest that systemic diphenhydramine use should be limited to increase the effectiveness of conventional therapies. Clinicians should have fewer reservations about recommending the use of loratadine concurrent with dry eye treatments based on the findings of this trial.

## Figures and Tables

**Table 1:** Descriptive results for all the outcome measures.

	PRT Test (mm)		TBUT (s)		IOP (mmHg)	
	OD	OS	OD	OS	OD	OS
Mean	21.83	21.02	8.65	8.70	15.01	14.77
Median	23.00	21.00	8.50	9.00	15.00	15.00
St Dev	5.85	5.94	2.81	2.96	3.89	3.75
Minimium	7	5	3	4	9	9
Maximum	35	37	23	21	21	21

PRT = phenol red thread, TBUT = tear break-up time, IOP = intraocular pressure.
